# The Role of FDG-PET in the Evaluation of Hidradenitis Suppurativa: A Systematic Review

**DOI:** 10.3390/jcm12175491

**Published:** 2023-08-24

**Authors:** Sahithi Talasila, Eric M. Teichner, Robert C. Subtirelu, David H. Xiang, Cyrus Ayubcha, Thomas Werner, Abass Alavi, Mona-Elisabeth Revheim

**Affiliations:** 1 Sidney Kimmel Medical College, Thomas Jefferson University, Philadelphia, PA 19107, USA; sxt561@students.jefferson.edu (S.T.); eric.teichner@gmail.com (E.M.T.); 2 Department of Radiology, Hospital of the University of Pennsylvania, Philadelphia, PA 19104, USA; robert.subtirelu@pennmedicine.upenn.edu (R.C.S.); tom.werner@pennmedicine.upenn.edu (T.W.); abass.alavi@pennmedicine.upenn.edu (A.A.); 3 Harvard Medical School, Harvard University, Boston, MA 02115, USA; dxiang@hms.harvard.edu (D.H.X.); ayucyrus@sas.upenn.edu (C.A.); 4 Department of Epidemiology, Harvard Chan School of Public Health, Boston, MA 02115, USA; 5 The Intervention Centre, Division of Technology and Innovation, Oslo University Hospital, 0424 Oslo, Norway; 6 Institute of Clinical Medicine, University of Oslo, 0317 Oslo, Norway

**Keywords:** hidradenitis suppurativa, non-invasive imaging techniques, fluorine-18 fluorodeoxyglucose, FDG-PET/CT, systemic inflammation, diagnosis, treatment follow-up, SAPHO syndrome

## Abstract

Hidradenitis suppurativa (HS) is a chronic skin disorder characterized by nodules, comedones, and sinus tracts that often leave prominent scarring. In recent years, non-invasive imaging techniques have been used to assess the inflammatory activity, vascularization, and treatment response of lesions. Specifically, fluorine-18 fluorodeoxyglucose positron emission tomography/computed tomography (FDG-PET/CT) scans may aid in identifying systemic inflammation in patients with HS, improving diagnosis. Inflamed hypermetabolic tissues exhibit a greater uptake of FDG due to increased glucose uptake and vascularity. A systematic review was conducted to summarize the utility of nuclear imaging techniques in the diagnosis and treatment follow-up of HS. PubMed, Scopus, and ScienceDirect databases were utilized for relevant articles discussing the utility of PET scans in managing HS. A total of 51 citations were identified in the initial search. Following the review of titles, abstracts, and duplicates, 43 articles were excluded, leaving a total of eight articles for analysis. Data were extracted from each article, encompassing the number of patients, imaging techniques employed, and final results. An analysis of the data demonstrated that FDG-PET showed evidence of identifying subclinical lesions of the disease, improving the visualization of HS, and providing an objective method of assessing severity.

## 1. Introduction

Hidradenitis suppurativa (HS) is a chronic inflammatory skin disorder characterized by recurrent nodules, abscesses, comedones, and sinus tracts that often leave prominent scarring in the axilla, groin, perianal, inframammary, and intermammary folds [1,2,3]. HS is more prevalent in women than men and affects up to 4% of the global population, with an estimated annual cost of $6500 per patient [4]. In one study, nearly half of the patients indicated that they could not afford the dressings and wound care supplies they would prefer in terms of both type and quantity [4].

Additionally, HS negatively impacts quality of life, as the lesions can be painful, disfiguring, and malodorous, hindering activities of daily living [5,6,7]. Specifically, one study demonstrated that said features of HS can result in significant impairments that commonly exceed other dermatoses, including psoriasis, atopic dermatitis, and acne vulgaris. Due to the severity of HS, significant psychological distress and psychiatric comorbidity may be concomitantly observed with the disease. Patients with HS endure a compromised quality of life, battling excruciating pain, recurrent abscesses, and chronic inflammation. The condition’s physical and emotional toll often leads to limited mobility, social isolation, and a profound impact on their overall well-being [8]. Unfortunately, treatment courses are often partially efficacious, and many patients are left with the aforementioned sequalae [8].

Although the exact pathophysiology of HS is unclear, the prevailing theory is that the condition is caused by the follicular occlusion of the folliculopilosebaceous unit, followed by rupture and inflammation [9,10,11]. Furthermore, a mutation in the γ-secretase enzyme that leads to the failure of keratin degradation has been linked to HS [12,13]. Risk factors include obesity, hormonal changes experienced during the postpartum and premenopausal states, stress, family history, and environmental factors such as smoking and mechanical friction [14]. Shaving or waxing, as well as friction from tight clothing, can aggravate inflammation by further disrupting hair follicles [14,15].

HS is a chronic condition characterized by inflammation, pain, pus formation, tissue damage, and scarring. Immune cells and cytokines play a significant role in the development of HS [16]. Keratin fibers and other debris from tissue destruction and scarring activate Toll-like receptors (TLRs) on macrophages and dendritic cells, leading to the production of the pro-inflammatory cytokines, tumor necrosis factor alpha (TNF-α), interleukin (IL)-17, and IL-1β [17]. The NLRP3 (nucleotide-binding domain, leucine-rich family pyrin domain containing 3) inflammasome is the best-characterized inflammasome and is activated by the release of material from follicles, leading to the production of IL-1β and neutrophil involvement [18]. HS lesions have reduced levels of IL-22, leading to an increased production of IL-10 due to increased IL-1β [1,19,20]. This intricate interplay between immune cells and cytokines creates a self-sustaining cycle of inflammation, causing recurrent pain, purulence, tissue damage, and scarring in HS patients [21].

These findings emphasize that comprehensive management is crucial in treating HS, with various treatments available depending on the severity of the condition [22]. Mild cases are treated with topical clindamycin and dapsone, while stages 1 and 2 HS involve combining rifampin with either oral clindamycin or minocycline. These antibiotics function to reduce inflammation, infections, and new lesions in HS. Prolonged antibiotic regimens are often used to control HS; however, this strategy may be inducing antibiotic resistance. One study found that lesions of HS patients on prolonged antibiotics grew significantly more antibiotic-resistant bacteria. These results underscore the importance of antibiotic stewardship when managing HS [23]. Alternatively, hormonal treatments such as oral contraceptives, spironolactone, and finasteride have also been used as adjuncts in severe disease or in mild-to-moderate presentations. As increased levels of androgens cause the folliculopilosebaceous gland to secrete more sebum, the mechanism of hormone therapy to treat HS functions by balancing testosterone and 5-dihydrotestosterone (5-DHT) levels [23]. Severe cases may require treatments like adalimumab, intravenous carbapenems, isotretinoin, laser therapy, and surgical excision. Adalimumab is a monoclonal antibody that targets TNF-alpha, blocking the inflammation that contributes to abscess, inflammatory nodules, and draining tunnels. Carbapenems function as broad-spectrum antibiotics, and isotretinoin inhibits keratinization and sebaceous gland function, resulting in smaller glands that are less likely to be clogged. Surgical interventions for HS encompass procedural approaches like laser therapy and minor surgical procedures, including incision and drainage or deroofing. A more extensive surgical option for severe disease would be wide local excision [23]. Laser Speckle Contrast Analysis (LASCA) is an innovative tool with diverse applications in dermatology and dermatosurgery. It measures speckle contrast to assess local blurriness in images, linked to tissue blood perfusion. LASCA effectively estimates skin microcirculation and aids in HS evaluation. It detects imperceptible foci and post-operative outcomes, including ischemic necrosis and lesion area assessment for surgical precision [4,23].

The diagnosis of HS is primarily a clinical one; however, histopathology is the gold standard for a definitive diagnosis [22]. Clinical diagnosis is made by the modified Dessau definition, which assesses the type of lesions, their location, and the patient’s medical history [23,24]. Specifically, three diagnostic criteria must be present [25]. First, there must be the presence of deep-seated painful nodules or other characteristic lesions, which can resemble abscesses, bridged scars, draining sinus, or post-inflammatory, open, tombstone double-ended comedones. Second, the lesions must appear in one or more of the predilection areas, including the axillae, inframammary and intermammary folds, groin, perineal region, or buttocks. Lastly, the disease must be chronic and recurrent, with at least two recurrences over a six-month period [25].

Other factors, including a family history of HS, recurrent uncharacteristic lesions, and the absence of pathogenic microbes, may also support the diagnosis [26]. The clinical presentation of painful lesions in the predilection areas, along with their recurrent and chronic nature, is usually sufficient for a self-reported diagnosis with a high degree of accuracy [27]. However, an observation period may be necessary to confirm the diagnosis in cases where there is no history of recurrence and chronicity, with a delay in diagnosis not exceeding six months [28].

As such, a more objective method for the diagnosis of HS is necessary. In recent years, non-invasive imaging techniques have grown in popularity for the assessment of the extent, inflammatory activity, vascularization, and treatment response of HS lesions. Imaging methods can also help prevent misdiagnosis and reduce diagnostic delays when HS is suspected [29]. However, there are limited publications on the subject.

Fluorodeoxyglucose positron emission tomography (FDG-PET) measures the metabolic activity of the cells in body tissues, allowing for the detection of inflammation, cancer, and infection [30,31]. While FDG-PET imaging is primarily used in the field of oncology, it has proven useful in other medical areas of cardiac and brain imaging. FDG-PET is known for its high sensitivity in detecting cellular inflammation in tissues and early signs of atherosclerosis [32]. Specifically in dermatology, FDG-PET has been used to ascertain systemic and vascular inflammation in psoriasis, detecting inflammatory lesions in the skin, blood vessels, joints, and liver [33]. Tissues that are hypermetabolic and inflamed exhibit a greater uptake of FDG compared to surrounding tissues, due to increased glucose uptake and vascularity. This distribution of FDG throughout the body allows clinicians to identify hypermetabolic tissues, as well as inflammatory and infectious conditions [29].

As chronic inflammation is commonly associated with systemic diseases, such as psoriasis, it is hypothesized that utilizing FDG-PET/CT scans may aid in identifying systemic inflammation in patients with HS [33]. In many HS patients, PET has incidentally detected clinical and subclinical lesions using FDG as the radiotracer. Although imaging methods have tremendous potential in HS diagnosis and management, few publications regarding radiology and nuclear medicine in this condition exist [33]. This review aims to summarize the utility of nuclear imaging techniques with emphasis on PET in the diagnosis and treatment follow-up of HS.

## 2. Materials and Methods

A systematic review was conducted to summarize the utility of nuclear imaging techniques in the diagnosis and treatment follow-up of HS. PubMed, Scopus, and ScienceDirect databases were utilized for relevant articles discussing the utility of PET scans in managing HS. The search strategy employed a combination of keywords, including “Hidradenitis Suppurativa”, “PET”, “nuclear imaging”, and “radiology” A total of 51 citations were identified in the initial search. Two independent reviewers assessed the titles and abstracts of these citations for relevance. Articles meeting the inclusion criteria were selected for full-text review. Studies included those that discussed the use of PET scans in managing HS, were published in English peer-reviewed journals, and had publication dates ranging from January 1985 to March 2023.

## 3. Results

**Figure 1:**
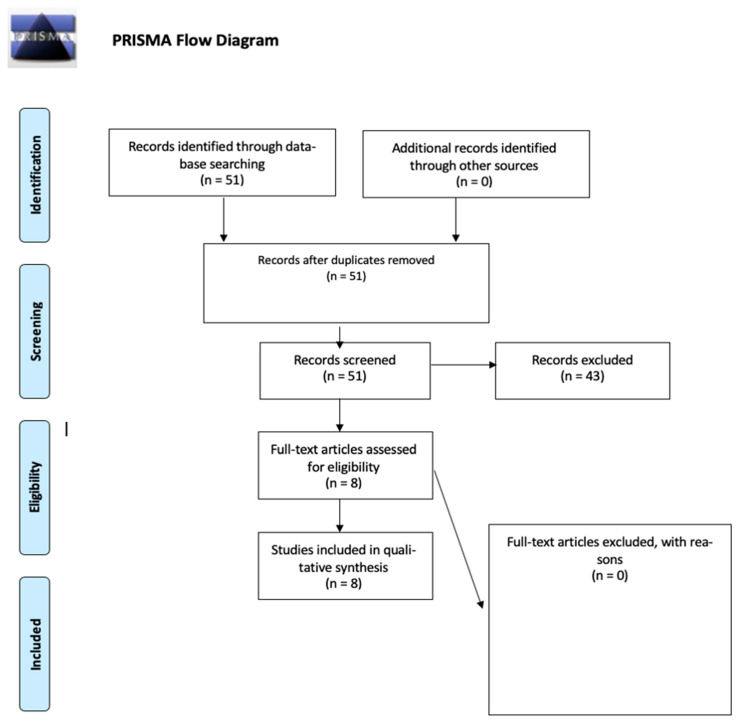
PRISMA flow diagram: The presented diagram illustrates the progression of information during the various stages of a systematic review. It outlines the count of identified records, both included and excluded, along with the rationales for exclusions.

Following the review of titles, abstracts, and duplicates, 43 articles were excluded, leaving a total of eight articles for analysis (Figure 1). Data were extracted from each article, encompassing the number of patients, imaging techniques employed, and final results. A narrative synthesis of the findings was conducted to summarize the utility of FDG-PET in diagnosing and monitoring the treatment of HS. To our knowledge, no other radiotracers have been utilized in HS analyses.

In summary, one article presented a case–control study involving 32 HS patients aged 18 years and older, which identified multiple HS foci on FDG-PET scans in clinically unexpected locations. Consequently, the authors found that FDG-PET scans in HS permit a more precise determination of disease burden, allowing clinicians to design more effective treatment plans [34]. Five articles featured case reports on the incidental detection of HS using FDG-PET scans while investigating malignant metastases. These case reports emphasized the capacity of FDG-PET scans to detect HS and demonstrated that detected lesions require further evaluation through biopsy to exclude occult malignancy [24,33,34,35,36]. The last two articles comprised comprehensive reviews on the usefulness of various radiological techniques in diagnosing and managing HS. The first review determined that CT imaging was considered inferior to PET imaging for HS; however, PET had limitations in visualizing anatomic localization and lesion extensions [37]. The second review concluded that there was no apparent advantage in imaging HS lesions with FDG-PET/CT scans, as these scans can result in false positives when attempting to identify metastatic cancer [38]. Multimodality imaging, i.e., employing a combination of imaging techniques, offers a solution to overcome the limitations of individual technologies. Our literature review determined that all current case reports of HS detected using hybrid imaging selected PET/CT as their modality of choice.

## 4. Discussion

### 4.1. Case–Control Study

In a case–control study involving 32 HS adult patients (Table 1), along with age- and sex-matched controls, numerous HS foci were detected on FDG-PET scans in clinically unexpected locations. Unlike in psoriasis, PET/CT scans did not identify systemic inflammation. Consequently, FDG-PET scans in HS facilitated a more accurate identification of disease burden, as demonstrated by the 84.4% (27/32) of patients in the study that had subclinical cutaneous inflammatory foci detected. This enables clinicians to plan for more effective treatment. Thus, FDG-PET is valuable for the prognostication of HS through its usefulness in mapping disease burden, particularly in subclinical cutaneous sites, as well as for detecting functional changes due to disease that precede anatomical changes [30].

### 4.2. Case Reports

In a case report of a 35-year-old man with classical Hodgkin’s lymphoma and a history of HS, FDG-PET scans displayed an unexpectedly intense uptake in the superficial subcutaneous tissues of the abdomen, pelvis, and chest wall. However, the radiological abnormalities corresponded with the clinical sites of HS involvement, providing the first documentation of HS on an FDG-PET scan [24]. This sparked further interest in evaluating the utility of FDG-PET for HS detection.

Similarly, in a case report of a 54-year-old man with metastatic small-cell lung cancer and a history of HS, FDG-PET scans revealed an increased uptake in the axillary, periscrotal, and perianal regions, which were clinically confirmed to be HS. Subsequent histopathological evaluation via biopsy confirmed the HS diagnosis [33]. FDG-PET/CT accurately detected both subclinical and clinical sites of HS involvement.

In another case report, a 64-year-old man with left-vocal-cord squamous-cell carcinoma was referred for an FDG-PET/CT scan. The scan showed uptake in the vocal cord malignancy and in subcutaneous foci in the right axilla, right buttocks, and scalp, corresponding to known HS lesion locations. This emphasizes the need to consider HS as a potential red herring that may be mistaken for metastases, as well as the utility of FDG in HS detection [34]. However, biopsy is necessary to confirm the diagnosis and differentiate HS from malignancy, depending on the clinical scenario.

In a unique case report of a 56-year-old man with a history of adenocarcinoma and HS, the patient was evaluated for lesions in the right axilla, which had become purulent and increased in size, initially diagnosed as HS. An FDG-PET scan revealed that the patient’s exophytic red nodules in his right axilla were metastases of adenocarcinoma, later confirmed through biopsy. This marked the first study to detect cutaneous metastases initially thought to be HS [35], highlighting the utility of FDG-PET scans in differentiating between HS and cutaneous malignancy.

In another case report of a 62-year-old man with recurrent abscesses in his bilateral buttocks for three years, he was diagnosed with HS after a doctor’s visit and underwent pelvic CT, which revealed a 10 cm abscess in the subcutaneous tissue. An FDG-PET/CT scan was then performed to determine the origin and condition, revealing an increased FDG uptake due to both HS and malignancy. Consequently, immunohistochemistry stains and biopsy were required to distinguish between the HS lesion and mucinous adenocarcinoma of the perineum. This case highlights the ability of FDG-PET scans to detect HS and demonstrates the need for further evaluation via biopsy to rule out occult malignancy [36].

### 4.3. Comprehensive Reviews

One review analyzed 63 studies concerning the use of various radiological techniques in managing HS. The authors concluded that providers were advised to employ ultrasonography for monitoring lesion progression and pharmaceutical treatment efficacy, as well as for accurate disease staging. To assess deep-seated disease manifestations, both CT and MRI were recommended, with MRI offering superior resolution and greater accuracy in depicting margin delimitation and radiologic features such as content and tunnel aperture. Although fistulography and mammography can be used to detect HS, they were most effective for assessing lesions. Imaging reports for HS have increased in recent years, leading the authors to suggest incorporating imaging techniques into routine clinical practice for HS patients.

Three case reports were retrieved from a search on FDG in cancer patients who incidentally had HS. It should be noted that these PET/CT examinations were not performed to detect HS, and the hypermetabolic subcutaneous lesions identified were not indicative of metastases but rather were consistent with HS. Additionally, it is important to consider the patient’s medical history when evaluating an atypical distribution of hypermetabolic foci to avoid misdiagnosis. Infection, sarcoidosis, surgery, and minor trauma such as intramuscular and subcutaneous injections can all lead to an increased metabolism in subcutaneous tissue. CT imaging is deemed inferior to PET imaging for HS, although PET has limitations in visualizing anatomic localization and lesion extensions. Thus, combining CT with PET imaging can facilitate HS visualization, and scintigraphy is capable of detecting small lesions with a full-body scan. Providers may use both PET/CT and PET/MRI as useful preoperative techniques to assess the extent of the lesion, detect subclinical lesions, and identify other complications, such as SAPHO syndrome (synovitis, acne, pustulosis, hyperostosis, and osteitis) associated with HS. However, no standardized method currently exists for identifying subclinical lesions. The evidence powering this review comprises 63 studies discussing HS and various radiologic imaging techniques, and includes randomized controlled trials, systematic reviews, books, observational studies, case reports, and case series [37].

Another review article that focused on imaging techniques in HS analyzed 55 studies concerning the use of ultrasound, MRI, PET, and CT for the diagnosis, management, and treatment of HS. Ultrasound (US) and MRI are the two main imaging techniques currently employed in HS patients, with MRI having a narrower range of use. US is well-studied and has improved the staging, pre-operative planning, and disease monitoring in HS patients. Almost any HS lesion can be effectively imaged by US, except for severe subcutaneous lesions. However, these sinus tract networks are most effectively visualized with the high contrast resolution of MRI. The application of medical infrared thermography (MIT) to HS patients is a recent innovative development that shows promise for the superficial detection of HS lesions before and during surgery. The authors of this specific study indicate that PET and CT imaging have incidentally detected HS lesions but have virtually no utility for the intentional imaging of HS [38]. For example, a limitation of FDG-PET/CT imaging, is that the inflammation of the HS areas may result in an increased FDG uptake, leading to false positives when clinicians are seeking to identify metastatic cancer. Thus, this review article concluded that there was no foreseeable benefit to imaging HS lesions with FDG-PET/CT scans, as they have shown no indication of being helpful. The evidence powering this review consists of 55 studies discussing HS and various radiologic imaging techniques, and includes randomized controlled trials, systematic reviews, books, observational studies, case reports, and case series [38].

Despite these limitations, FDG-PET/CT can be utilized to provide a more objective identification of HS through enhanced visualization. For example, early functional changes can be detected before anatomical changes occur, which allows for an earlier initiation of treatment. In one particular study, it was found that FDG-PET/CT scans were able to detect subclinical skin lesions in up to 84.4% of HS patients [33]. Additionally, this imaging technique allows for the whole body to be imaged in one session, which helps clinicians determine the optimal timing for treatment initiation or modification based on the disease burden. For instance, patients with extensive subclinical lesions (and a high disease burden) may require more aggressive treatment and a longer duration of systemic therapy. Mapping disease burden can also be useful for prognostication and early identification of individuals with potentially more severe or refractory disease courses. In HS patients, FDG-PET/CT scans did not reveal any evidence of systemic inflammation, unlike in patients with psoriasis, where FDG uptake was observed in multiple organs. The authors speculate that systemic inflammation may develop only after a prolonged period. The study participants were mostly young, and follow-up with repeat FDG-PET/CT scans would be necessary to evaluate the potential development of systemic inflammation [33].

There have been previous reports linking HS with various types of malignancies. In one study, it was found that almost 10% of patients had high FDG focal uptake over the thyroid glands, and two of these patients were confirmed to have papillary thyroid carcinoma. It was observed that papillary thyroid cancer was the most commonly reported malignancy, which corresponds with the current literature on this subject. This study also reported a 50% increased risk of all types of cancer in HS patients when compared to matched controls, with a higher risk for nonmelanoma skin cancer, buccal cancer, and primary liver cancer [33]. The high percentage of thyroid nodule detection and its association with malignancy in this small group of HS patients indicate a significant correlation that requires further investigation, specifically where FDG-PET/CT scans could detect malignancies and HS in high-risk patients.

Additional research into radiologic techniques has provided valuable information regarding HS [39,40,41]. Ultrasonography has been used to study healthy hair follicles and revealed elongated, low-echogenic structures in a regular pattern within the dermis at an angle of around 45° to 60° to the surface of the skin [42]. In HS, follicles have a larger diameter and distorted shape, and hair shafts appear as bilaminar hyperechoic linear structures retained within pseudocysts, abscesses, or fistulas [43]. This can be useful in identifying children with HS, as they have a higher prevalence of retained hair tracts within fluid collections and fistulous tracts as compared to adults [44]. Ultrasonography can also be used to assess skin thickness, resistance, density, and vascularization, with blood flow being generally higher around fluid collections and fistulous tracts coexisting with slow arterial and venous flow areas [45]. Interestingly, even perilesional areas that appear healthy and unaffected by inflammation can be seen as affected by HS when scanned [46].

**Figure 2:**
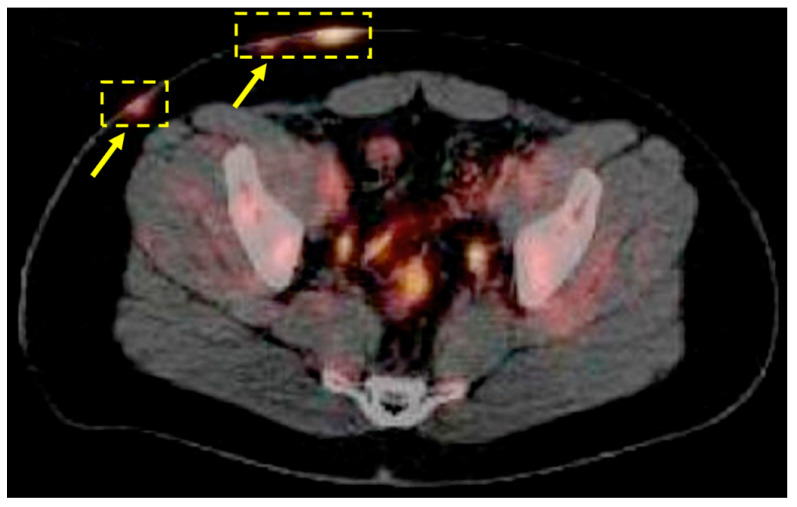
Positron emission tomography imaging of hidradenitis suppurativa (HS). 18-Fluorodeoxyglucose (FDG) was administered to visualize Hodgkin’s lymphoma in this hidradenitis suppurativa (HS) patient. There was substantial FDG uptake in the areas with HS lesions, inside of the dashed boxes, which resulted in the first documented case of positron emission tomography (PET) imaging of HS. Reproduced from Simpson et al., 2011 [32].

However, there are some limitations to using US imaging that PET scans can address. Distinguishing between small vasculature and developing sinus tracts, especially at the borders of HS lesions, is challenging for US imaging, which limits its effectiveness and accuracy in detecting the full extent of HS lesions [47,48]. Additionally, US imaging is operator-dependent and requires specialized training and experience in HS detection to achieve proficiency. Obtaining US images can also be time-consuming, making it challenging to incorporate it into clinical practice [49]. Moreover, HS patients may experience pain due to the physical contact required to obtain US images. Lastly, HS lesions can be deep-seated, and US imaging may not be able to penetrate the required depth, making it less useful, especially in larger patients [50]. FDG-PET scans can overcome these limitations and have a role in the diagnosis and management of HS patients, particularly in situations where US imaging is not feasible (Figure 2).

## 5. Conclusions

As mentioned in the current literature, performing FDG-PET scans in patients with cancer is useful in differentiating the HS processes from malignant processes. FDG-PET scans are useful in detecting subclinical lesions of the disease, improving the visualization of HS, and providing an objective method of assessing and diagnosing HS, aiding the management of the disease. Both hybrid imaging with PET/CT or PET/magnetic resonance imaging (MRI) could be used as a preoperative technique to assess the extent of the lesion and to identify other complications such as SAPHO syndrome (synovitis, acne, pustulosis, hyperostosis, and osteitis) associated with HS. An additional advantage offered by FDG-PET scans is an objective way of assessing disease burden that could be particularly helpful for the skin of patients of color. Although HS is prevalent among people of color, it is known that HS lesions can be more difficult to detect in darker skin tones, as the expected erythema can be difficult to see and lesion colors may present differently.

## Figures and Tables

**Table 1:** Demographics and outcomes of the studies included.

Article	Demographics/Subject Information	Modality	Summary Points	Recommendation on Utility (Yes or No)
Loo 2021 [30]	32 Hidradenitis suppurativa (HS) patients above 18, with age- and sex-matched controls	Fluorodeoxyglucose (FDG)—positron emission tomography (PET)/computed tomography (CT)	Numerous HS foci were detected on FDG-PET scans in clinically inapparent sites, especially in over nasal, mandibular, and scalp regions. However, PET/CT scan did not detect any systemic inflammation, unlike in psoriasis. Therefore, PET/CT scan may be useful to map a more accurate disease burden in HS patients.	Yes
Simpson 2011 [32]	35-year-old man with classical Hodgkin’s lymphoma	FDG-PET/CT	This is a reported case of a 35-year-old man with classical Hodgkin’s lymphoma who underwent a PET scan to evaluate his response to treatment. The scan revealed an unexpectedly high uptake in the superficial subcutaneous tissues of the abdomen, pelvis, and chest wall, which raised concerns but was later found to correspond to the areas affected by the man’s history of HS. This finding represents the first documented appearance of HS on a PET scan.	Yes
Ekmekcioglu 2020 [33]	54-year-old male patient with small-cell lung carcinoma	FDG-PET/CT	A literature report describes the case of a 54-year-old man who was referred for a follow-up PET/CT scan due to metastatic small-cell lung carcinoma. The scan detected an elevated F-18 FDG uptake in the axillary, periscrotal, and perianal regions, which was later identified as hidradenitis suppurativa. It was advised that a biopsy should be conducted to differentiate between hidradenitis suppurativa and metastatic disease.	Yes
Asamoah 2018 [34]	64-year-old man with a left-vocal-cord squamous-cell carcinoma	FDG-PET/CT	A review of the literature reports on a 64-year-old man who underwent an 18F-FDG-PET/CT scan due to left-vocal-cord squamous-cell carcinoma. The scan showed an increased uptake in the vocal cord tumor as well as subcutaneous foci in the right axilla, right buttocks, and scalp, which were already recognized as known locations of hidradenitis suppurativa skin lesions. The case emphasizes the importance of considering hidradenitis suppurativa as a possible cause of 18F-FDG incidental uptake, which can be misinterpreted as metastases, indicating the need for a careful interpretation of PET/CT findings.	Not Given
Baig 2022 [35]	56-year-old Hispanic man with a history of adenocarcinoma and hidradenitis suppurativa	FDG-PET	The individual under examination is a 56-year-old male of Hispanic origin who has previously been diagnosed with adenocarcinoma and hidradenitis suppurativa. The patient was assessed due to the appearance of purulent and enlarging lesions in the right axilla, initially believed to be a manifestation of HS. However, a PET scan revealed that the exophytic red nodules present in the right axilla were in fact metastases of adenocarcinoma, thus differentiating between HS and malignancy.	Yes
Kim 2020 [36]	62-year-old man had recurrent abscesses in his bilateral buttocks for 3 years	FDG-PET/CT	For three years, a 62-year-old man experienced recurring abscesses in both his buttocks. After seeking medical attention, he was diagnosed with hidradenitis suppurativa, and subsequently underwent a pelvic CT scan which revealed the presence of a 10 cm abscess in the subcutaneous tissue. Further investigation was carried out using a PET scan to ascertain the origin and condition, which indicated an increase in FDG uptake. This uptake was attributed to both hidradenitis suppurativa and malignancy.	Yes
Gutfilen-Schlesinger 2021 [37]	Review of the current literature	FDG-PET/CT and PET/MRI	A study of 63 radiological technique studies for managing HS recommends ultrasonography for monitoring lesion progression and staging, CT and MRI for assessing deep-seated disease (MRI more accurate), and underused PET for locating subclinical lesions and monitoring treatment progress.	Not over other radiologic techniques
Elkin 2020 [38]	Review of the current literature	FDG-PET/CT and PET/MRI	This review of 55 studies concluded that ultrasound and MRI are the primary imaging techniques for HS diagnosis, management, and treatment. Ultrasound has improved disease monitoring, planning, and staging, while MRI has limited but valuable use. CT and PET have limited utility for intentional imaging of HS, and are rarely mentioned in the literature.	Not over other radiologic techniques

Note: Data were extracted from each article, encompassing the number of patients, imaging techniques employed, and final results.
